# Multifaceted role of *Moringa oleifera* leaf extract as antimicrobial, growth enhancer and mitigator of salt stress in tomato seedlings

**DOI:** 10.1186/s12870-025-07149-7

**Published:** 2025-08-27

**Authors:** Reda E. Abdelhameed, Doaa A. Galilah, Rabab A. Metwally

**Affiliations:** 1 Botany and Microbiology Department, Faculty of Science, Zagazig University, Zagazig, Egypt; 2 Botany Department, Faculty of Science, Mansoura University, Mansoura, Egypt

**Keywords:** Tomato, Antimicrobial, Salinity, Biopriming, Moringa extract, Phosphatases, Stability index

## Abstract

Research on *Moringa oleifera* L.‘s potential applications as a dietary supplement, medicinal herb and plant growth enhancer under biotic and abiotic stresses has increased recently. Our study aimed to investigate the phytochemical screening of Moringa leaf aqueous extract (MLAE) and to determine its antimicrobial activity against pathogenic bacteria and fungi. Additionally, it investigated how MLAE biopriming affected tomato seedlings exposed to varying salinity levels. When three different MLAE concentrations (100, 250, and 500 µL) were tested against pathogenic microorganisms, an inhibitory zone appeared around the extract, demonstrating that MLAE could inhibit the growth of *Bacillus* sp., *B. cereus*,* Klebsiella* sp., *Pseudomonas* sp., *P. aeruginosa*, *Acinetobacter* sp., *Fusarium* sp1, *Fusarium* sp2, *F. oxysporum*. Moreover, aiming to mimic the negative effects of salt stress (0, 10, 25, 50 and 100 mM NaCl) on tomato seedlings, tomato seeds were soaked for 12 h in 0.1 and 0.2% of MLAE stock solution in addition to water (water priming, WP) and control unsoaked seeds. The results displayed that both germination and seedling growth traits were adversely affected by different NaCl concentrations, while seed soaking with MLAE alleviated the adverse effects of this stress. Most specially, MLAE significantly promoted plumule and radicle growth of tomato seedlings by the increases in seedling fresh and dry weights (mg) (246 ± 6.51a and 13.2 ± 0.35a) as compared with NaCl-free treatment (160 ± 4.233ef and 8.7 ± 0.23 fg). MLAE and WP application, under different salt concentrations, reduced the malondialdehyde content while increasing the antioxidant and phosphatase activities. Particularly, 0.2% MLAE caused the highest increase in protein and proline levels (2.54 mg/g fwt and 0.761 µmols/g fwt), followed by 0.1% MLAE and WP. Hence, this study affirmed the potency of MLAE as an antimicrobial agent against some pathogens and as a mitigator of salt stress in tomato seedlings suggesting its dual role in plant protection and stress tolerance as an eco-friendly and cost-effective alternative for integrated crop management.

## Introduction

Several antimicrobial agents with pharmacological effects and therapeutic utility that can be obtained from natural sources have been explored previously [1–3]. The study of plants and their pharmacological properties for the treatment of illness is known as phytotherapy. For instance, *Moringa oleifera* L., popularly referred to as the “tree of life” [4], is the most prevalent species of the Moringa genus. It is a source of several phytochemical substances, including glucosinolates, and possesses antibacterial activity [1]. Numerous active ingredients found in *M. oleifera* L. include triterpenoids, alkaloids, tannins, flavonoids, and saponins that have antibacterial properties [3, 5, 6]. Moreover, other metabolites such as zeatin, cytokinin, potassium, calcium, protein, ascorbate, vitamin A and vitamin C in *M. oleifera* L. play a crucial part in promoting plant growth and protecting it against environmental stresses [7].

One of these environmental stressors that negatively impacts crop production is salinity which is a major issue in sustainable food production, especially in arid and semi-arid countries [8–10]. The two crucial stages that are the most vulnerable to salinity in crop establishment are seed germination and seedling development [11] through osmotic stress, ion-specific phytotoxic effects (Na^+^ and Cl^−^), and oxidative stress [12, 13], which may result in detrimental impact on the seed’s physiological and biochemical processes. Elevated amounts of Na^+^ and Cl^−^ can hinder seeds’ ability to absorb water, preventing germination, or they can induce delayed and decreased emergence of seedlings, leading to irregular establishment which diminishes agricultural yields [13, 14].

As a result, effective strategies are required to manage this stress, especially for crops that are moderately or very sensitive to salt. Several methods have been employed recently to improve crops’ resistance to salt stress; one such technique is seed priming, which performs well and is advantageous for many field crops, as it promotes uniform and speedy emergence, high seedling vigor, and higher yields. It is also easy, inexpensive, and low-risk [13, 15]. Pre-treatment, particularly at the seed stage, has the potential to establish long-lasting stress reduction for present and future generations. It has been discovered that using plant biostimulants, *M. oleifera* L. extract in particular, holds great promise in sustainable agriculture due to its dual functionality. As a natural antimicrobial agent, it helps control various plant pathogens, reducing the need for synthetic pesticides. Simultaneously, it acts as a potent biostimulant, enhancing plant growth, improving stress tolerance, and promoting overall crop productivity through its rich content of phytohormones, antioxidants, and nutrients [7, 16]. MLAE, as a foliar spray or seed amendment enhances seed germination and seedling vigor, stimulates root development, and enhances photosynthetic efficiency under favorable and adverse conditions [17, 18]. By increasing antioxidant enzyme activity and raising phenols, flavonols, sugars, and osmolytes concentration, the application of MLAE lessens the effects of heat, salt, drought and heavy metals stresses. This diminishes the amount of reactive oxygen species (ROS), electrolyte leakage, and lipid peroxidation [16]. Similar to this, Moringa extract was employed by Abdel Latef et al. [7] to lessen salt stress in *Trigonella foenum-graecum*.

Tomatoes are the main dietary source of vitamin C, potassium, folate, vitamin K and antioxidants linked to several health benefits, including a decreased risk of heart disease and cancer [19]. According to Souri and Tohidloo [20], salinity has a major impact on tomato plants by reducing seed germination and slowing seedling growth. In this perspective, the development of tomato seeds’ ability to germinate under salinity is crucial. The current study aims to investigate the antimicrobial activity of MLAE against pathogenic bacteria and fungi and to elucidate the probable role of MLAE priming in improving salinity tolerance in tomatoes by examining germination parameters and some physiological characteristics related to the salinity-tolerant response.

## Materials and methods

### Moringa leaf aqueous extract (MLAE)

#### Collection and preparation of MLAE

This experiment was conducted at the Mycology Laboratory of the Botany and Microbiology Department, Faculty of Science, Zagazig University. Fresh *M. oleifera* L. leaves were obtained from a local herbal store on the 10th of Ramadan City, El-Sharkia Governorate, Egypt. The leaves were washed well with water to remove any adhering dirt, then air-dried for 48 h and ground into a fine powder in a home grinder. The powder was passed through a sieve and extracted by weighting out 100 g of air-dried *M. oleifera* L. leaf powder and dissolved in 1000 mL of warm water at 50 °C for 24 h in a conical flask stoppered with rubber cork, with frequent shaking in an incubator. The obtained aqueous extract of *M. oleifera* L. leaves was centrifuged at 3000 rpm for 10 min using centrifuge (Gemmy PLC-03). The resultant extract was kept in a sterile glass container and stored at 4 °C in a refrigerator until use for qualitative phytochemical screening and the duration of the experiments.

#### Phytochemical screening of MLAE

The MLAE was screened to identify its active phytochemicals such as phenolic, flavonoids, alkaloids, tannins, saponins, coumarins, steroids and glycosides, using standard procedures as described by Sofowora [21] and Iqbal et al. [22]. Most especially, for the phenols test, 2 mL of distilled water followed by a few drops of 10% ferric chloride was added to 1 mL of MLAE. A blue/green color formation indicated the presence of phenols. The flavonoids test was done by combining 1 mL of MLAE with 1 mL of 2 N NaOH solution. The appearance of a yellow precipitate confirmed its presence. Tannins were detected by mixing equal parts distilled water and MLAE, stirring, and adding a few drops of FeCl_3_. The presence of tannins was confirmed by the production of a green precipitate. The presence of alkaloids was demonstrated by the turbidity after adding Wagner’s reagent to 3 mL of 1% concentrated HCl and 3 mL of MLAE in a steam bath. In order to identify coumarins, 1 mL of MLAE was combined with 1 mL of 10% NaOH and its existence was established by the emergence of a yellow color. For saponins, 5 mL of distilled water and 5 mL of MLAE were heated in a test tube after being violently shaken and the production of stable foam confirmed its presence.

#### Estimation of total soluble protein, phenolic, flavonoids, free amino acids contents and total antioxidant capacity of MLAE

The total soluble protein in MLAE was calculated using the Lowry et al. [23] technique, furthermore, its total phenolic contents was assessed at 765 nm using Jindal and Singh’s [24] Folin Ciocalteu reagent-based test. The total flavonoid contents were determined at 510 nm according to Zou et al. [25]. According to Lee and Takahashi [26] protocol, the amino acid contents of MLAE were determined. The total antioxidant capacity was examined using the phosphomolybdenum procedure [27]. One mL of the extract was combined with 3 mL reagent solution (28 mM sodium phosphate, 0.6 M H_2_SO_4_ and 4 mM ammonium molybdate). The mixture was heated to 95 °C for 90 min, cooled, and the absorbance at 695 nm was recorded.

### MLAE as antimicrobial agent

#### Tested microorganisms

The tested pathogenic bacteria in our experiment were obtained from the Bacteriology Lab., Faculty of Science, Zagazig University, and were used to assess the antimicrobial effect of MLAE. It included *Bacillus* sp. and *B. cereus* as Gram-positive bacteria and *Klebsiella* sp., *Pseudomonas* sp., *P. aeruginosa* and *Acinetobacter* sp. as Gram-negative bacteria. Furthermore, MLAE was assessed against some pathogenic fungi such as *Fusarium oxysporum*,* Fusarium* sp1., *Fusarium* sp2., *Aspergillus flavus* and *A. niger* that were originally isolated from the soils of El-Sharkia governorate, Egypt.

#### Antimicrobial assay of MLAE

The antimicrobial activity of MLAE was individually achieved against each isolated bacteria and fungus *via* the well diffusion method [28–30] using 100, 250, and 500 µL/well. For bacteria, nutrient agar medium was prepared and autoclaved at 121 °C for 15 min. The medium was added to sterile glassy Petri dishes (90 mm in diam.), allowed to solidify for 30 min, and then swabbed with 18-h-old cultures (25 µL) of bacterial suspension of each tested bacteria (10^7^ CFU/mL) on plates. For fungi, a potato dextrose agar (PDA) medium was used, sterilized and transferred into sterilized petri dishes (90 mm in diam.). After solidification, 100 µL inoculum of each tested fungal culture suspension containing approximately 10^6^–10^7^ conidia/mL was swabbed by spreading the inoculums using sterile cotton swabs horizontally and vertically to get uniform microbial growth on the Petri plates. Wells of 9 mm were punched with a sterile cork borer on the agar plate, which was then filled with three different volumes of MLAE (100, 250 and 500 µL) after using a syringe filter (0.22 μm) to filter MLAE. Negative controls were prepared using the water that was used to dissolve MLAE. The bacterial or fungal plates were then left for 2 h in a refrigerator, after that they were incubated for 18–24 h at 37 °C for bacterial plates or 4 days at 28 ± 2 °C for fungal plates. Each test was carried out in triplicate. Using a caliper, the diameters of the MLAE-inhibited growth zones against the tested bacteria or fungi were measured in millimeters.

### MLAE as a growth enhancer for tomato seedlings under salt stress

#### Preparation of Murashige and Skoog (MS) medium with different NaCl concentrations

The culture medium was prepared by weighing 4.4 g MS/L and 3% sucrose. Five salt levels (0, 10, 25, 50, and 100 mM NaCl) were applied to the prepared MS media, and then 0.8% agar was added to the media with stirring and heating. The media were then dispersed in 100 mL screw-capped glass jars, 25 mL per jar and autoclaved at 121 °C and 15 psi for 20 min.

#### Seeds, priming and surface sterilization

Tomato (*Solanum lycopersicum* L.) seeds were obtained from the Agricultural Research Center, Giza, Egypt. Uniform seeds were split into four treatment groups: the first group was left unprimed as a control treatment, the second group was soaked in water (water priming, WP) treatment, the third treatment group was soaked in a 0.1% MLAE stock solution, and the fourth treatment group was soaked in 0.2% MLAE stock solution. Seeds were soaked for 12 h at 25 °C in the dark, then left to dry in air for 6 h. To ensure sterilization, each group of seeds was surface sterilized with 70% (v/v) ethanol for 30 s, then in 30% (v/v) NaOCl with frequent shaking, and rinsed in sterile distilled water several times.

#### Sowing and incubation

To assess the impact of soaking treatments on seed resilience to salt stress, the sterilized seeds were sown in glass jars on MS media having different levels of NaCl under an aseptic environment in a laminar flow cabinet that had a horizontal flow of sterile air. All glass jars were kept in an incubator with a 16-h photoperiod and a controlled temperature of 27 °C. The treatments were set up in a 4 × 5 factorial with three replicates for each of the five salt levels (0, 10, 25, 50, and 100 mM NaCl) and four soaking treatments (control, WP, 0.1 and 0.2% MLAE).

#### Germination, seedling growth and sampling

Every day, the number of germinated seeds was observed and after 14 days, the final germination percentage (GP) was calculated (GP = (number of seeds germinated/total seed number) x 100). In each treatment, three normal seedlings were chosen randomly, and their radicle and plumule lengths were measured and reported in centimeters (cm). The length of the seedlings was also measured using the same three typical seedlings. The seedlings’ fresh weight (fwt) was measured and their dry weight (dwt) was recorded in milligrams (mg) after keeping for a whole day at 70 °C in a hot air oven. The seedling vigor and salt tolerance indices were computed using the GP, seedling length, and seedling dwt data. According to Abdul-Baki and Anderson [31] and Bouslama and Schapaugh [32], the seedling length vigor index (SLVI), seedling weight vigor index (SWVI) and salt tolerance index for each treatment were computed using the following formulas:

$$\mathrm{SLVI}\;=\;\left[\mathrm{seedling}\;\mathrm{length}\;\left(\mathrm{cm}\right)\;\times\;\mathrm{GP}\;\left(\%\right)\right]$$

$$\mathrm{SWVI}\;=\;\left[\mathrm{seedling}\;\mathrm{length}\;\mathrm{dwt}\;\left(\mathrm{mg}\right)\;\times\;\mathrm{GP}\;\left(\%\right)\right]$$

$$\mathrm{SSI}\;=\;\mathrm{SS}\;/\;\mathrm{SC}$$

*SSI is the seedling stability index, and SS and SC are the total dry matter (mg per seedling) under saline stress and non-stress conditions.

Also, the germination rate index (GRI) and the mean germination time (MGT) were calculated according to Maguire [33] and Labouriau [34].

$$\mathrm{GRI}\;=\;\mathrm\Sigma\;\left(\mathrm{ni}/\mathrm{ti}\right)$$

$$\mathrm{MGT}\;=\;\left(\mathrm{\Sigma niti}\right)\;/\;\mathrm{\Sigma ni}$$

*ni is the number of germinated seeds on a given day, and ti is the time in days from the sowing day (0).

#### Seedling water status

The measurements of the water content (WC), relative water content (RWC) and water saturation deficit (WSD) were made by Barr and Weatherley [35] technique. After measuring the seedlings’ fwt, they were cut into small pieces and floated in deionized water for 4 h before their turgid weight (twt) was determined. Following a 24-hour drying period at 80 °C in an oven, samples were weighed once again (dwt). The following formulas were utilized to estimate WC, RWC, and WSD:

$$\mathrm{WC}\;=\;\left(\mathrm{fwt}\;-\mathrm{dwt}\right)/\mathrm{fwt}\;\times\;100$$

$$\mathrm{RWC}=\;\left(\mathrm{fwt}\;-\mathrm{dwt}\right)/\left(\mathrm{twt}\;-\mathrm{dwt}\right)\;\times\;100$$

$$\mathrm{WSD}\;=\;100-\mathrm{RWC}$$

#### Malondialdehyde (MDA) content

MDA, a lipid peroxidation byproduct, was quantified utilizing a Hodges et al. [36] modified approach. Fresh seedling samples (0.2 g) were milled in 5 mL of 0.1% (w/v) trichloroacetic acid (TCA). An aliquot of 1 mL from the supernatant was added to 4 mL of 0.5% (w/v) thiobarbituric acid (TBA) in 20% (w/v) TCA after centrifugation at 6000 rpm for 5 min. Samples were heated for 30 min at 90 °C. Following that, the reaction was stopped in an ice bath, and the supernatant’s absorbance was measured at 532 nm and adjusted for non-specific turbidity by deducting the absorbance at 600 nm *via* a UV-visible spectrophotometer [RIGOL, Model Ultra-3660]. The MDA content was estimated using the following formula, and expressed as nmol MDA g^−1^ fwt:

MDA (nmol g ^− 1^ fwt) = [(A532 − A600) × V × 1000/ɛ] × fwt

*ɛ is the specific extinction coefficient (155 mM cm^−1^), V is the volume of the crushing medium, A_532_ and A_600_ are the absorbance at 600 nm and 532 nm wavelengths.

#### Protein and proline contents

The total soluble protein content and antioxidant enzyme activities of fresh tomato seedlings were measured after being ground in 10 mL of cold 50 mM K-phosphate buffer (pH 7) containing 1 mM ethylenediaminetetraacetic acid (EDTA). The resultant solution was centrifuged at 6000 rpm for 15 min. For protein, 1 mL of the mixture was subjected to shaking for 10 min after adding alkaline copper sulfate reagent and Folin’s reagent and placed in an incubator for 30 min, after that the absorbance was read at 700 nm [23]. The total protein concentration was calculated as mg/g fwt using bovine serum albumin as a reference. For proline extraction, known fwt of tomato seedlings were ground in 3% sulphosalicylic acid [37]. In test tubes, 2 mL of freshly prepared acid-ninhydrin solution was added to 2 mL of the supernatant and 2 mL of glacial acetic acid. The tubes were incubated in a water bath at 90 °C for 30 min and the reaction was terminated in an ice bath and was extracted with 4 mL of toluene, and the absorbance was recorded at 520 nm and its content was determined as follows:

$$\:\:\mathbf{P}\mathbf{r}\mathbf{o}\mathbf{l}\mathbf{i}\mathbf{n}\mathbf{e}\:\mathbf{c}\mathbf{o}\mathbf{n}\mathbf{c}\mathbf{e}\mathbf{n}\mathbf{t}\mathbf{r}\mathbf{a}\mathbf{t}\mathbf{i}\mathbf{o}\mathbf{n}\:(\varvec{\upmu\:}\mathbf{m}\mathbf{o}\mathbf{l}\mathbf{e}\mathbf{s}/\mathbf{g}\:\mathbf{f}\mathbf{w}\mathbf{t})=\frac{(\varvec{\upmu\:}\mathbf{g}\:\mathbf{p}\mathbf{r}\mathbf{o}\mathbf{l}\mathbf{i}\mathbf{n}\mathbf{e}/\:\mathbf{m}\mathbf{L}\:\times\:\:4\:\mathbf{m}\mathbf{L}\:\mathbf{t}\mathbf{o}\mathbf{l}\mathbf{u}\mathbf{e}\mathbf{n}\mathbf{e})}{115.5\:\mathbf{x}\:\:\mathbf{g}\:\mathbf{f}\mathbf{w}\mathbf{t}\:\mathbf{o}\mathbf{f}\:\mathbf{s}\mathbf{a}\mathbf{m}\mathbf{p}\mathbf{l}\mathbf{e}\:}$$

*115.5 is the molecular weight of proline.

#### Phospholytic enzymes (Acid and alkaline phosphatases)

Following maceration in 0.1 M borate buffer (pH 8.5) of a known fwt of germinated tomato seedlings, the homogenate was centrifuged for 10 min at 6000 rpm. P-nitrophenylphosphate (PNPP) was used as a substrate to quantitatively assess the amount of soluble phosphatases [38]. One enzyme activity unit was defined as 1nmol of PNP/min.

#### ROS-scavenging enzymes

By measuring the rise in absorbance at 470 nm using pyrogallol as the substrate, peroxidase (POX) activity was measured following Chance and Maehly’s [39] protocol. According to Beyer and Fridovich [40] and Nakano and Asada [41], the activities of polyphenol oxidase (PPO) and ascorbate peroxidase (APX) were measured and expressed as U g^−1^ fwt.

### Statistical data analysis

Duncan’s test and analysis of variance (ANOVA) were used to analyze the means at a 5% significant level. The SPSS program (Statistical Package for Social Science V16.0) was used to do a one-way ANOVA on the data. Data in tables and figures refer to mean values (*n* = 3) ± standard error. Pearson correlation coefficients, Hierarchical clustering analysis and principal component analysis (PCA) graphs between different treatments and parameters were created using Past 4.03 (PAleontological STatistics) programme.

## Results and discussion

### Phytochemical screening and quantitative analysis of phyto-constituents of MLAE

**Fig. 1:**
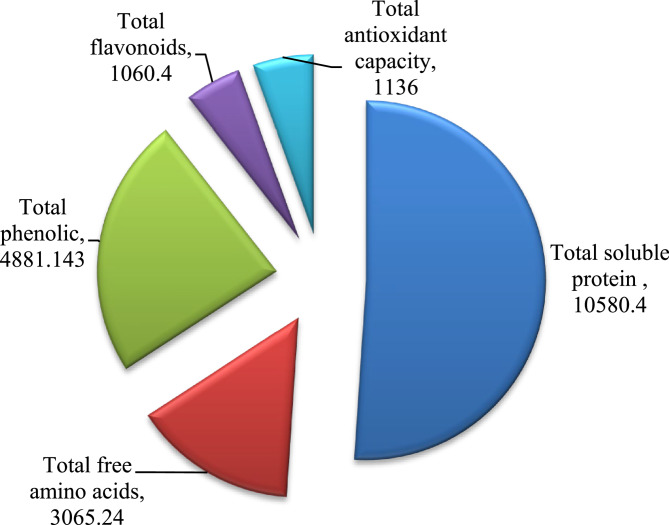
Quantitative analysis (µg/mL) of phytochemical constituents (total phenolic, flavonoids, free amino acids, soluble protein and antioxidant capacity) in Moringa crude extract

Basically phytochemical screening is used to reveal the chemical constituents of the plant extract. Results in Table 1 showed that the MLAE is a good source of secondary metabolites such as phenolic, alkaloids, flavonoids, saponins, and proteins with high quantities as shown in Fig. 1. Our findings are supported by Yasmeen [42] and Yasmeen et al. [43] who confirmed that MLAE is an excellent source of active ingredients due to the presence of phenols, proteins, zeatin, ascorbates, carotenoids, antioxidants, and other essential nutrients. According to Patel et al. [44], the phytochemical screening of *M. oleifera* L. exhibited the presence of alkaloids, flavonoids, saponins, sterols and tannins in both aqueous and ethanolic extracts of leaves. Furthermore, the study of Fahal et al. [45], revealed the presence of alkaloids, flavonoids, saponins, sterols and tannin in the extract of *M. oleifera* L.

**Table 1:** Qualitative phytochemical screening of MLAE

Phytochemicals	MLAE
Phenolic	**++**
Flavonoids	**+**
Saponins	**+**
Alkaloids	**+**
Coumarins	**+**
Proteins	**+**
Tannins	**+**
Glycosides	**-**

Highly present ‘++’, present ‘+’, absent ‘−’

### Antimicrobial activity of MLAE

**Fig. 2:**
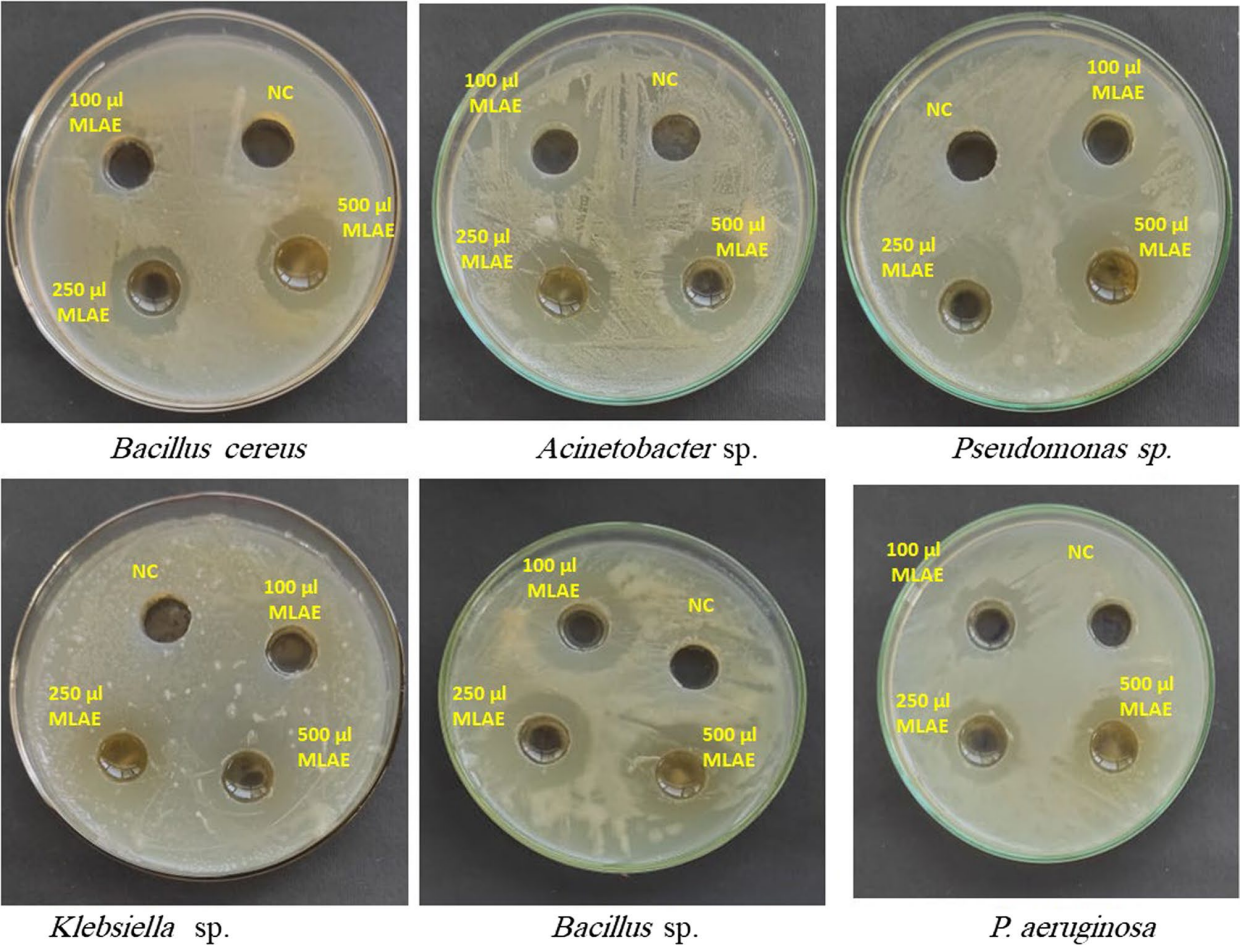
Antibacterial activity of different concentrations (100, 250 and 500 µL) of MLAE against some pathogenic bacteria (*Bacillus* sp., *B. cereus*,* Klebsiella* sp., *Pseudomonas* sp., *P. aeruginosa* and *Acinetobacter* sp.). *NC: Negative control

*M. oleifera* L. is regarded as one of the novel approaches to the management of pathogenic fungi and bacteria. The current study demonstrated that the antimicrobial activity of MLAE was assessed using the well diffusion method, as shown in Fig. 2; Tables 2 and 3. MLAE demonstrated a pronounceably superior antibacterial action against *Bacillus* sp., *B*. *cereus*,* Klebsiella* sp., *Acinetobacter* sp., *Pseudomonas* sp. and *P. aeruginosa;* where their corresponding inhibition zone diameters were 24.0 ± 0.63, 14.6 ± 0.38, 19.6 ± 0.52, 16.6 ± 0.44, 28.0 ± 0.74, and 19.6 ± 0.52 mm, (respectively), at 500µL MLAE. Moreover, MLAE showed a relatively obvious antifungal effect against *Fusarium oxysporum*,* Fusarium* sp1. and *Fusarium* sp2. with their individual diameter zones of inhibition recorded at 35.0 ± 0.92, 32.0 ± 0.84, and 23.3 ± 0.61, (respectively). However, no inhibitory action was determined for MLAE against *A. flavus* and *A. niger*. The results showed that the antimicrobial activity of MLAE increased with increasing concentration. The antimicrobial properties of MLAE could be attributed to its contents of phytochemical compounds, for instance, phenolic, flavonoid, polyphenols, flavonoids, other proteins, and peptides contents (Elhadi et al. 46), as previously reported in Table (2).

**Table 2:** Antibacterial activity of different concentrations of MLAE against some pathogenic bacteria

Bacteria	Zone of inhibition (mm)		
	100 µl MLAE	250 µl MLAE	500 µl MLAE
**Gram-negative** ***Klebsiella*** **sp.**	9 ± 0.23d	15.0 ± 0.39c	16.6 ± 0.44d
***Pseudomonas sp.***	21.6 ± 0.57a	23.3 ± 0.61a	28.0 ± 0.74a
***P. aeruginosa***	13.3 ± 0.352c	14.6 ± 0.38c	19.6 ± 0.52c
***Acinetobacter*** **sp.**	13 ± 0.34c	15.6 ± 0.41c	19.6 ± 0.52c
**Gram-positive** ***Bacillus*** **sp.**	20.3 ± 0.53b	21.3 ± 0.56b	24.0 ± 0.63b
***B. cereus***	10 ± 0.26d	11.6 ± 0.30d	14.6 ± 0.38e

*Values are presented as mean ± S.E of triplicate experiments. Mean values in the same column followed by the same lower-case letter are not significantly different according to ANOVA and Duncan tests at the 0.05 level of confidence

**Table 3:** Antifungal activity of different concentrations of MLAE against some pathogenic fungi

Fungus	Zone of inhibition (mm)		
	100 µl MLAE	250 µl MLAE	500 µl MLAE
***Fusarium oxysporum***	18.3 ± 0.48a	24.0 ± 0.63a	35.0 ± 0.92a
***Fusarium*** **sp1**	2.0 ± 0.05c	16.0 ± 0.42b	32.0 ± 0.84b
***Fusarium*** **sp2**	5.6 ± 0.14b	15.3 ± 0.40b	23.3 ± 0.61c
***Aspergillus flavus***	nd	nd	nd
***Aspergillus niger***	nd	nd	nd

*nd *No detection, *Values are presented as mean ± S.E of triplicate experiments. Mean values in the same column followed by the same lower-case letter are not significantly different according to ANOVA and Duncan tests at the 0.05 level of confidence

In addition, ascorbic acid, flavonoids, phenolic, and carotenoids, natural antioxidants are abundant in Moringa leaves [2, 46]; these active substances have an antibacterial effect and can overcome the problem of multidrug resistance [47, 48]. Our findings were consistent with Elhadi et al. [47]., Moyo et al. [49]., and Abdallah et al. [50] that MLAE had antimicrobial properties by inhibiting the growth of pathogenic bacterial strains. Likewise, Dhakad et al. [51] and van den Berg et al. [52] reported that *M. oleifera* L. is distinguished for having antifungal and antibacterial qualities. Numerous in vitro investigations have shown that variant extracts from various *M. oleifera* L. sections have inhibitory activity on Gram-positive bacteria such as *Staphylococcus aureus* and Gram-negative bacteria such as *Salmonella enterica*, *P. aeruginosa*, *K. pneumoniae*, and *Escherichia coli* [3, 50, 53, 54]. Additionally, Jayawardana et al. [55] revealed that pterygospermin, a chemical component with well-established antibacterial properties that readily splits into two molecules of benzyl isothiocyanate, is present in *M. oleifera* L. leaves [50]. Therefore, MLAE could be used as a promising strategy attributable to its antimicrobial properties against pathogenic microorganisms.

### MLAE pretreatment enhanced tomato seeds germination and seedling growth under salt stress

Salt stress resulted in an overall reduction of the GP and GR of tomato seedlings, as indicated by the decreases in plumule length, radicle length, seedling fwt as well as dwt (Table 4). This reduction was increased with the rise of salinity levels. There was a reduction in seedling length (28.8%) and seedling dwt (19.5%) in seeds subjected to extreme salt stress (50 mM) as compared to the NaCl-free treatment. Similarly, Abdalla et al. [13] and Metwally and Soliman [56] confirmed that germination and seedling parameters of soybean and tomato were critically reduced by salinity. It’s possible that under salt stress conditions, seeds develop an osmotically enforced “dormancy” that accounts for the drop in GR, especially under high NaCl stress. This could be a seed’s adaptive way of preventing germination under stressful situations [57]. Also, the slower GR of tomato seedlings may be attributed to the inhibition of cell division, elongation and enlargement caused by elevated salinity levels [13, 58]. Furthermore, high osmotic stress and ion toxicity are among the other factors [56].

**Table 4:** Effect of water priming (WP) and Moringa leaf aqueous extract (MLAE) priming on tomato seedling parameters in response to different NaCl concentrations

Priming	NaCl (mM)	Seedling fwt (mg)	Seedling dwt (mg)	Plumule length (cm)	Radicle length (cm)	Seedling total length (cm)
**Control**	0	160 ± 4.233ef	8.7 ± 0.23 fg	10 ± 0.26 cd	3.2 ± 0.085c	13.2 ± 0.349def
	10	145 ± 3.836gh	7.8 ± 0.21ij	10 ± 0.26 cd	3 ± 0.079c	13 ± 0.343efg
	25	132 ± 3.492hi	7.3 ± 0.19jk	8 ± 0.21f	3.1 ± 0.082c	11.1 ± 0.294 h
	50	100 ± 2.646j	7 ± 0.18k	6.4 ± 0.17 g	3 ± 0.079c	9.4 ± 0.248i
	100	-	-	-	-	-
**WP**	0	200 ± 5.29b	10.6 ± 0.28 cd	10.8 ± 0.29bc	3.8 ± 0.100b	14.6 ± 0.386bc
	10	160 ± 4.23ef	8.9 ± 0.24 fg	11 ± 0.29b	3.2 ± 0.085c	14.2 ± 0.376 cd
	25	150 ± 3.96 fg	8.2 ± 0.22ghi	9.5 ± 0.28de	3 ± 0.079c	12.5 ± 0.331 fg
	50	120 ± 3.17i	7.5 ± 0.19ijk	8.2 ± 0.25f	3 ± 0.079c	11.2 ± 0.296 h
	100	-	-	-	-	-
**0.1% MLAE**	0	240 ± 6.35a	11.6 ± 0.31b	11.5 ± 0.30ab	4 ± 0.105b	15.5 ± 0.410ab
	10	180 ± 476 cd	9.9 ± 0.26de	12 ± 0.26a	3 ± 0.079c	15 ± 0.397abc
	25	160 ± 4.23ef	8.6 ± 0.23fgh	10 ± 0.24 cd	4 ± 0.105b	14 ± 0.370cde
	50	140 ± 3.71gh	7.9 ± 0.21hij	9 ± 0.29e	3 ± 0.106c	12 ± 0.317gh
	100	-	-	-	-	-
**0.2% MLAE**	0	246 ± 6.51a	13.2 ± 0.35a	11.5 ± 0.31ab	4.5 ± 0.119a	16 ± 0.423a
	10	188 ± 4.97bc	11.1 ± 0.29bc	11 ± 0.29b	4 ± 0.105b	15 ± 0.396abc
	25	169 ± 4.47de	10.4 ± 0.27 cd	11 ± 0.29b	4 ± 0.105b	15 ± 0.397abc
	50	160 ± 4.23ef	9.2 ± 0.24ef	10 ± 0.26 cd	3 ± 0.079c	13 ± 0.345efg
	100	-	-	-	-	-

Mean values (*n* = 3) in the same column for each trait followed by the same lower-case letter are not significantly different according to ANOVA and Duncan tests at the 0.05 level of confidence

Most interestingly, water and MLAE priming had an obvious effect on tomato seedling growth (Table 4). MLAE significantly promoted plumule and radicle growth of tomato seedlings as seen by the increases in seedling fwt and dwt (mg) (246 ± 6.51a and 13.2 ± 0.35a) as compared with NaCl-free treatment (160 ± 4.233ef and 8.7 ± 0.23 fg). MLAE (0.2%) was the most effective in promoting germination parameters, followed by 0.1% MLAE and WP. Salinity levels significantly reduced the seedling dwt of unprimed seeds; however, this reduction was completely countered by water or MLAE seed priming. This demonstrated that water or MLAE seed priming were more resilient to the rise in salinity.

Additionally, the use of MLAE alone significantly improved the characteristics of tomato seedlings and also diminished the detrimental effects of salinity. Based on Rady and Mohamed [58] and Rady et al. [59] researches, these results validated the stimulatory properties of moringa extract. MLAE application for salt-stressed *Phaseolus vulgaris* increased development through the water use efficiency [60]. Also, moringa extract priming of wheat and maize seeds influenced the metabolite fraction, which resulted in higher biomass and growth [18, 43]. Plant extracts in both fresh and dry forms have been shown to stimulate growth by upregulating the activity of several enzymes involved in critical metabolic pathways [61]. Furthermore, MLAE treatments improved shoot or leaf K^+^, decreased uptake of unwanted Na^+^ and/or Cl^−^, raised levels of plant secondary metabolites and stimulated the plant defense system that rapid the growth of seedlings [42, 62].

### Effect of MLAE pretreatment in tomato seedling vigor index and stability index under salt stress

The SLVI of tomato seedlings was drastically reduced with the rise of salt levels (Table 5), which evidenced the negative effect of salinity on the elongation of tomato seedlings (Table 4). This result agreed with Abdalla et al. [13] on soybean seedlings under salt stress. Seed priming with water, or MLAE, has a beneficial effect under salt stress, which results in higher SLVI as compared to unprimed ones. Under 50 mM salt stress, SLVI was significantly greater for MLAE (1170) followed by WP seeds (918.4) and lower for unprimed seeds (689.02). Additionally, Table (5) showed that seed priming caused higher SWVI and SSI under salt stress conditions. A similar observation was found by Oliveira et al. [63] on melon seedlings. The SVI and SSI had been used as tolerance indices to evaluate the salinity effect on seedling growth [64]. During periods of extreme salt stress a decrease in SVI was observed. This could be because salinity prevents seedlings from growing during their early stages. The SSI of tomato seedlings under 10 mM NaCl increased from 0.897 (under the control condition) to 1.276 (under 0.2% MLAE) (Table 5). Our findings demonstrated that water priming greatly raised the SVI and GP. This was consistent with Shabbir et al. [65].

**Table 5:** Response of salt-stressed tomato seed to priming with MLAE in relation to the germination traits (GP: germination percent, SLVI: seedling length Vigor index, SWVI: seedling weight Vigor index, SSI: seedling stability index, GRI: germination rate index, MGT: mean germination time)

Priming	NaCl (mM)	GP (%)	SLVI	SWVI	SSI	GRI (seed/day)	MGT (day)
**Control**	0	100 ± 2.645a	1320 ± 34.9d	290 ± 7.67e		6.375 ± 0.168de	6.368 ± 0.168 cd
	10	100 ± 2.66a	1300 ± 34.3d	260 ± 6.87 fg	0.897 ± 0.0237 fg	5.542 ± 0.146f	6.529 ± 0.173bcd
	25	80 ± 2.11d	888 ± 23.49 h	194.675.15i	0.839 ± 0.0222gh	3.958 ± 0.1047 h	6.923 ± 0.183ab
	50	73.3 ± 1.93e	689.02 ± 18.22i	171.03 ± 4.53j	0.805 ± 0.0213 h	1.917 ± 0.051j	7.429 ± 0.196a
	100	-	-	-	-	-	-
**WP**	0	100 ± 2.64a	1460 ± 38.62bc	353.33 ± 9.34c		7.375 ± 0.195bc	6.143 ± 0.163 cd
	10	100 ± 2.63a	1420 ± 37.56b	296.67 ± 7.85e	1.023 ± 0.0271 cd	6.875 ± 0.182 cd	6.250 ± 0.165 cd
	25	90 ± 2.38b	1125 ± 29.76 fg	246 ± 6.51 h	0.943 ± 0.0249de	6.083 ± 0.161e	6.333 ± 0.167 cd
	50	82 ± 2.17 cd	918.4 ± 24.29 h	205 ± 5.42i	0.862 ± 0.0228fgh	2.750 ± 0.073i	7.158 ± 0.189a
	100	13 ± 0.34f	-	-	-	-	-
**0.1% MLAE**	0	100 ± 2.64a	1550 ± 41.01ab	386.67 ± 10.23b		8.125 ± 0.215a	6.000 ± 0.159d
	10	100 ± 2.64a	1500 ± 39.68abc	330 ± 8.71d	1.138 ± 0.0301b	7.875 ± 0.208ab	6.045 ± 0.1599 cd
	25	90 ± 2.38b	1260 ± 33.33de	258 ± 6.83 fg	0.989 ± 0.0262cde	7.458 ± 0.197b	6.095 ± 0.161 cd
	50	88 ± 2.33bc	1056 ± 27.94 g	231.73 ± 6.13 h	0.908 ± 0.024efg	4.875 ± 0.128 g	6.414 ± 0.196 cd
	100	13 ± 0.34f	-	-	-	-	-
**0.2% MLAE**	0	100 ± 2.66a	1600 ± 42.33a	440 ± 11.64a		8.125 ± 0.215a	6.000 ± 0.158d
	10	100 ± 2.63a	1500 ± 39.71abc	370 ± 9.78bc	1.276 ± 0.0337a	7.375 ± 0.195bc	6.143 ± 0.163 cd
	25	95 ± 2.51a	1425 ± 37.7c	329.33 ± 8.71d	1.195 ± 0.0316b	6.583 ± 0.174de	6.211 ± 0.164 cd
	50	90 ± 2.38b	1170 ± 30.95ef	276 ± 7.31ef	1.057 ± 0.0279c	4.208 ± 0.111 h	6.615 ± 0.175bc
	100	6 ± 0.16 g	-	-	-	-	-

Mean values (*n* = 3) in the same column for each trait followed by the same lower-case letter are not significantly different according to ANOVA and Duncan tests at the 0.05 level of confidence

The GRI of tomato seedlings decreased significantly **(**Table 5) with increasing salt concentration, ranging from 5.542 ± 0.146f (at 10 mM NaCl) to 1.917 ± 0.051j (at 50 mM NaCl). On a similar level, Gharoobi et al. [66] reported that the GRI and GP of maize seeds were considerably lowered with the rise of the osmotic potential level. This decrease may be attributed to the lower capacity of water uptake by the seeds with high salt. Also, data in Table 5 showed that the MGT increased with increasing salt concentration while priming with water or MLAE decreased this time significantly.

### MLAE regulated physio-biochemical characteristics in NaCl-exposed tomato seedlings

**Fig. 3:**
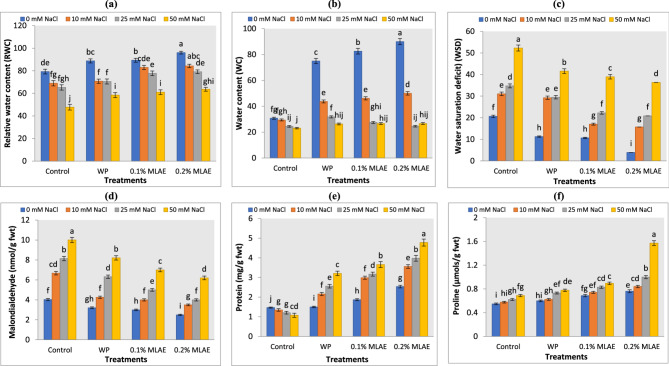
Effect of water priming (WP) and moringa leaf aqueous extract (MLAE) priming on (**a**): relative water content (RWC), (**b**): water content (WC), (**c**): water saturation deficit (WSD), (**d**): malondialdehyde, (**e**): protein and (**f**) proline content of tomato seedlings under different NaCl concentrations. Bars followed by the same letters, are not significantly different by ANOVA and Duncan tests at the 0.05 level of confidence. Data refer to mean values (*n* = 3) ± standard error

The RWC and WC were significantly reduced in tomato seedlings subjected to salt stress (Fig. 3a and b). RWC decreased by 17.7 and 39.8% in seedlings exposed to 25 and 50 mM NaCl as compared to those under non-saline conditions. Furthermore, WSD (Fig. 3c) increased significantly with the salt, and this was in accordance with Metwally and Soliman [56] and Metwally and Abdelhameed [67] in tomato and fenugreek. Plants grown in saline conditions are subjected to physiological drought as Na^+^ and Cl^−^ ions bind water that is required for the seedlings and lead to a reduction in the water content [68]. Conversely, all priming treatments caused a significant increase in RWC and WC (Fig. 2), with a subsequent decrease in WSD compared to the unprimed ones. MLAE applications maintained optimum tissue water status, membrane stability, and enhanced antioxidant levels as Yasmeen et al. [42] and Rehman et al. [62] reported.

The changes in MDA in tomato that reflect the degree of oxidative stress damage to lipid membranes [69] are presented in Fig. 3d. Salt stress resulted in an increase in MDA that corroborated with Mostofa et al. [70] Abdelhameed and Metwally [71] and Abdelhameed et al. [72] due to plant losing its ability to restrict ROS [73]. However, all priming treatments showed a reduction in this parameter. Priming with water caused a non-significant decrease as compared with unprimed seeds. Moreover, priming with MLAE (0.1 and 0.2%) resulted in a lower MDA content, in which 0.2% MLAE had a significant lowest MDA among the treatments. MLAE reduced MDA generation by shielding the biological membrane from the oxidative effects of harmful free radicals, which may be related to the MLAE’s phenols and other active metabolites content (Fig. 1 and Table 1). As seen in *P. vulgaris* and bean plants under salt stress, MLAE was thought to stop excessive membrane leakage and stabilize structures suffering lipid peroxidation [60, 74]. Our findings also in line with Yasmeen et al. [43], who found that salt tolerance was improved by phenol accumulation through MLAE by mediating ROS scavenging and preserving membrane stability.

According to Farhangi-Abriz and Torabian [75], salinity frequently causes the production of osmotic stress, which limits plants’ access to water and causes cellular dehydration, a loss of turgidity and eventually death. In order to counteract this osmotic stress, plants accumulate osmolytes, which decrease osmotic potential and maintain cellular turgor and water retention [73, 76]. Proline, as an osmolytes, keeps membranes stable and stops breaking down of proteins and enzymes under stress and plays an important role in ROS scavenging [77]. Regardless of the degree of salt, Figure (3e and f) showed that tomatoes priming with water and MLAE increased their proline and total soluble protein contents in a substantial way (*p* < 0.05). Soluble protein content in tomatoes significantly (Fig. 3e) decreased after NaCl treatment (% of decrease were 17.7 and 26.5 after 25 and 50 mM NaCl salt treatment alone compared with those under non-saline conditions). This is due to the fact that high Na^+^ levels affect plants by disrupting protein synthesis [11, 78]. Most interestingly, 0.2% MLAE caused the highest increase in protein and proline levels (2.54 mg/g fwt and 0.761 µmols/g fwt), followed by 0.1% MLAE and WP (Fig. 3). The increase with MLAE may serve as an energy reservoir or possibly as an osmotic potential adjuster in plants undergoing salinity because in vitro protein synthesis systems are dependent on physiological K^+^ and are inhibited by Na^+^ and Cl^−^. Our results are also in harmony with Abdel Latef et al. [7], who reported an accumulation of proline in *T. foenum-graecum* plants. Bourgne et al. [79] reported that priming increases the solubilization of seed storage proteins like the B-subunit of the 11-S globulin in *Beta vulgaris*. The buildup of other osmolytes such as glycine betaine and soluble sugars is another approach to overawed osmotic stress induced by salt stress [80].

### MLAE pretreatment elicited ROS scavenging system under salt stress condition

**Fig. 4:**
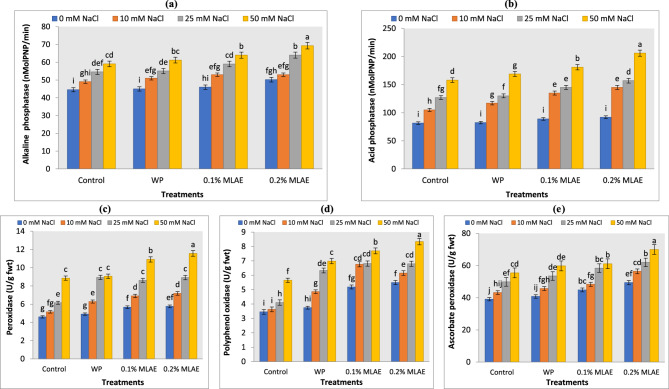
Effect of water priming (WP) and moringa leaf aqueous extract (MLAE) priming on the activity of alkaline phosphatase (**a**), acid phosphatases (**b**) and on the activity of antioxidant enzymes (**c**) peroxidase, (**d**) polyphenol oxidase and (**e**) ascorbate peroxidase of tomato seedlings under different NaCl concentrations. Bars followed by the same letters, are not significantly different by ANOVA and Duncan tests at the 0.05 level of confidence. Data refer to mean values (*n* = 3) ± standard error

To gain further insight into the enzymatic changes during stress conditions, we studied phosphatases and antioxidant enzyme activities in tomato seedlings. Results indicated that NaCl significantly induced an increase in alkaline and acid phosphatases in tomato seedlings (Fig. 4a and b). Furthermore, their activities were significantly increased with MLAE under both non-saline and saline stress conditions, compared to their corresponding controls. Our findings are in line with Sharma et al. [81] in Pearl millet. Acid phosphatase maintains a certain level of inorganic phosphate in plant cells under stress [82]. This could because stress limits development and impedes phosphate transport, which causes the cellular phosphatases to become active. Phosphatases modulate osmotic adjustment by the free phosphate absorption and releasing soluble phosphate from its insoluble constituents either inside or outside of cells. Ehsanpour and Amini [83] reported a rise in acid and alkaline phosphatases in alfalfa with salt stress, which might be due to the high resistance of the preexistent acid phosphatase to stress-induced degradation or stress-stimulated new acid phosphatase synthesis [84].

**Fig. 5:**
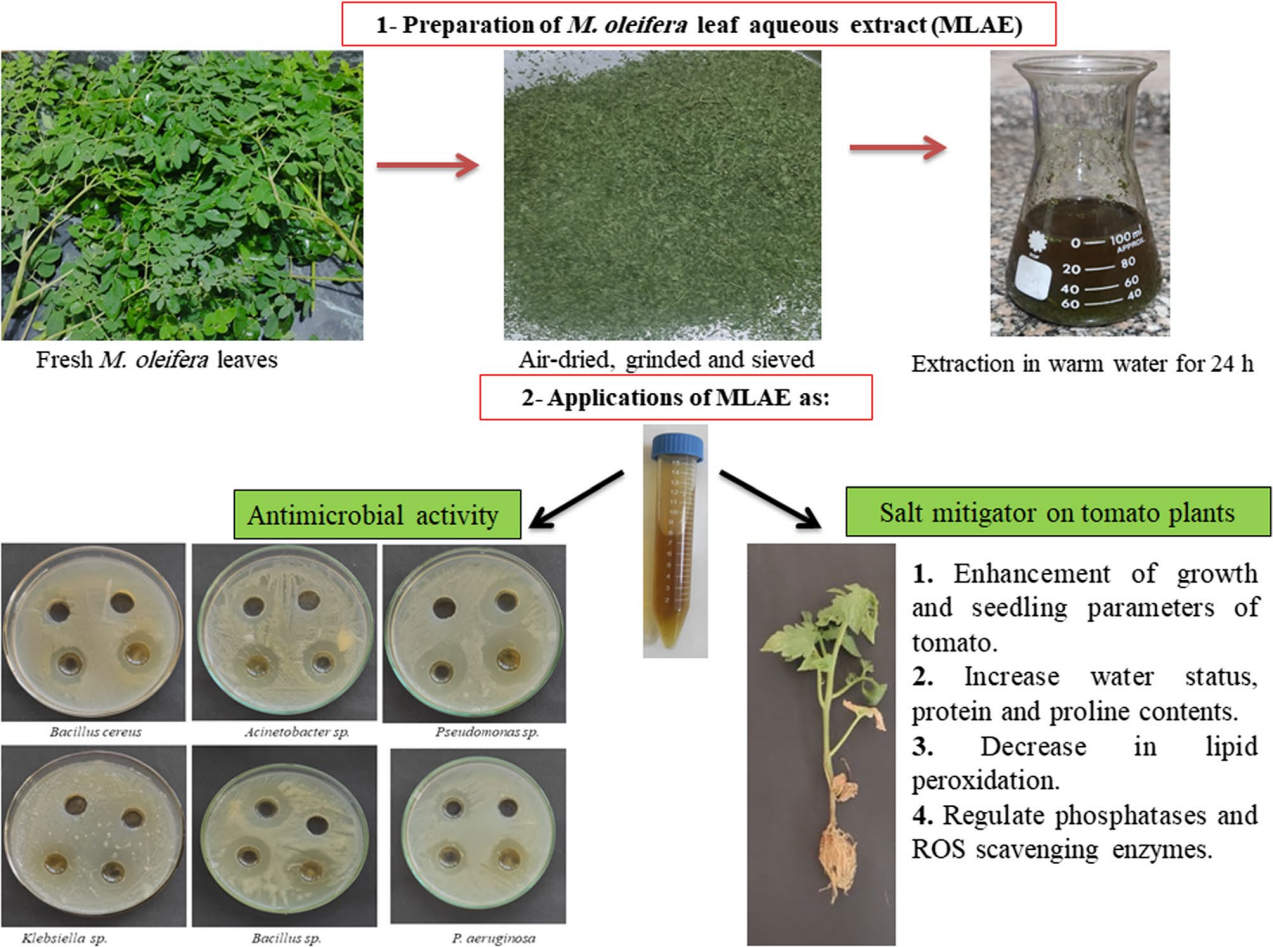
A diagrammatic illustration for MLAE preparation and application

**Fig. 6:**
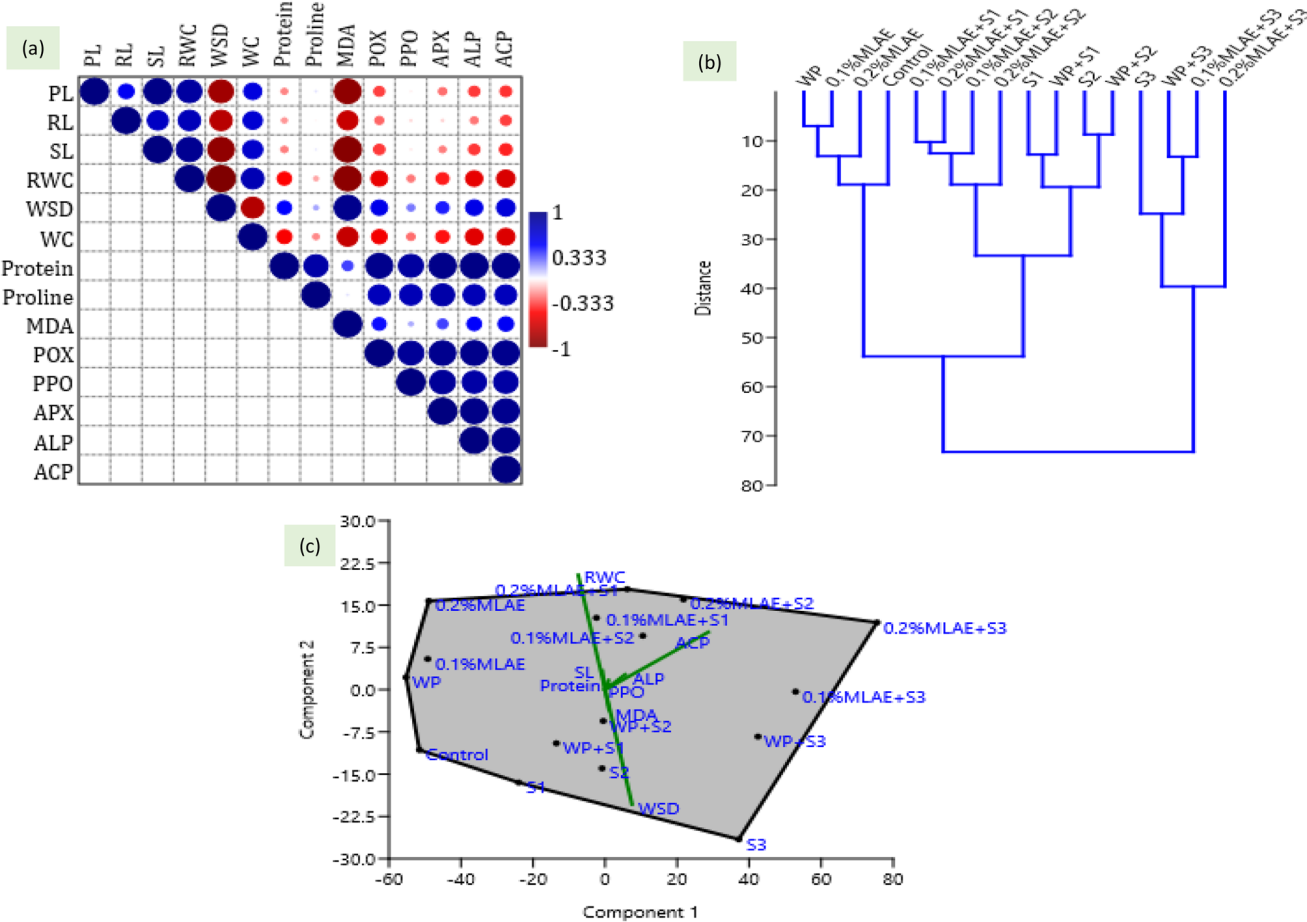
**a** Pearson correlation between different measured parameters, (**b**) Hierarchical clustering between different treatments and (**c**) Scatter plot of PCA analysis. S1, S2 and S3 represent 10, 25 and 50 mM NaCl

As a result of excessive ROS generation due to salt stress, plants normally cope with this oxidative damage by increasing the antioxidant enzyme activities [85], including POX, APX, and PPO, which increased slightly in tomato seedlings subjected to salt stress. MLAE application (0.1% and 0.2%) caused a further increase in their activities, followed by WP **(**Fig. 4c-e**)**. Our results demonstrated that MLAE alleviates the adverse effect of salinity by enhancing the production of POX, APX, and PPO, thereby providing strength to the antioxidant defense system in the removal of toxic ROS. These results are in accordance with Rady et al. [59] that MLAE is rich in some antioxidants, including ascorbic acid, phenols, proline, phytohormones, auxins, and cytokinins [74]. These metabolites may be absorbed by the seeds and supported the antioxidant system in seedlings and then in plants, which enables plants to overcome salinity. Furthermore, Abdel Latef et al. [7] discovered that foliar spraying by MLAE boosted POX activity, which raises lignin formation and related defensive chemicals that either directly or indirectly lessen damage. Collectively, Fig. 5 summarizes the preparation of MLAE and its application as antimicrobial and tomato salt mitigator. Figure 6 showed the data interrelationship between different parameters and treatments which confirmed the role of MLAE in alleviating salt stress in tomato.

## Conclusions

Our results proposed that MLAE could be used as a promising strategy due to its antimicrobial properties against pathogenic microorganisms and can be used as a priming agent to enhance the growth of tomatoes under salt stress. MLAE, at both concentrations, enhances germination parameters, which may increase water absorption that maintain water content in tomato seedlings under salt conditions. In addition, MLAE reduced the severity of salinity by improving total soluble protein content, proline, and enzymatic antioxidants and minimizing oxidative stress by lowering MDA content. Thus, it could be utilized in the agricultural sector as a biostimulant and eco-friendly strategy for mitigating salt stress in crops.

### Practical implications and scalability

The study highlights the prospective of MLAE as a natural, scalable, sustainable tool and low-input alternative for alleviating salinity stress in crops particularly for resource-limited farming systems. A thorough cost analysis comparing MLAE to conventional chemical treatments would help assessing its economic viability for large-scale farming. Furthermore, the commercial use of MLAE may involve navigating regulatory frameworks related to the registration of biostimulants, safety assessments, and quality control standards. Addressing all these practical and regulatory aspects will be critical for promoting the widespread adoption of MLAE in sustainable agriculture.

### Future directions

While the study provides convincing initial evidence of MLAE’s efficacy, further research is warranted to broaden its applicability. Future investigations should emphasis on appraising its performance across diverse crop species with varying genetic tolerances to salinity. It would also be beneficial to study the long-term effects of repeated MLAE applications on plant health, soil microbiota, and yield quality. Moreover, integrating MLAE with other stress mitigation strategies, such as microbial inoculants could offer synergistic benefits and pave the way for more resilient agricultural systems under saline conditions.

## Data Availability

All data generated or analyzed during this study are included in this published article.

## Abbreviations

APX: Ascorbate peroxidase
Dwt: Dry weight
Fwt: Fresh weight
GRI: Germination rate index
GP: Germination percent
MDA: Malondialdehyde
MGT: Mean germination time
MLAE: Moringa leaf aqueous extract
POX: Peroxidase
PPO: Polyphenol oxidase
ROS: Reactive oxygen species
RWC: Relative water content
SLVI: Seedling length vigor index
SWVI: Seedling weight vigor index
SSI: Seedling stability index
WC: Water content
WP: Water priming
WSD: Water saturation deficit
